# A β‐galactosidase activated near‐infrared fluorescent probe for tracking cellular senescence in vitro and in vivo

**DOI:** 10.1002/smo.20240062

**Published:** 2025-01-05

**Authors:** Tian Su, Ruijun Shen, Dengchu Tu, Xiaoyue Han, Xianzhu Luo, Fabiao Yu

**Affiliations:** ^1^ Key Laboratory of Emergency and Trauma Ministry of Education Key Laboratory of Haikou Trauma Key Laboratory of Hainan Trauma and Disaster Rescue The First Affiliated Hospital of Hainan Medical University Hainan Medical University Haikou China; ^2^ Engineering Research Center for Hainan Bio‐Smart Materials and Bio‐Medical Devices Key Laboratory of Hainan Functional Materials and Molecular Imaging College of Emergency and Trauma College of Pharmacy Hainan Medical University Haikou China; ^3^ Department of Chemistry Shanghai Engineering Research Center of Molecular Therapeutics and New Drug Development School of Chemistry and Molecular Engineering East China Normal University Shanghai China; ^4^ Ludwig Institute for Cancer Research University of Oxford Oxford UK

**Keywords:** β‐gal, cellular senescence, fluorescent probe, lysosomes, near‐infrared

## Abstract

Cellular senescence is a steady state of cell cycle arrest necessary to maintain homeostasis in organisms. However, senescent cells may cause senescence in neighboring healthy cells, inducing the onset of several diseases, such as inflammation, neurological disorders, and atherosclerosis. Therefore, early detection of cellular senescence is extremely important. β‐Galactosidase (β‐gal), as a critical marker of cellular senescence, can be monitored to facilitate early diagnosis of aging‐related diseases. Furthermore, β‐gal is mainly found in lysosomes, which have a pH value of about 4.5–5.5. Here, we developed a near‐infrared fluorescent probe (QMOH‐Gal) for tracking cell senescence in vitro and in vivo via the detection of β‐gal. In addition, the probe displayed high sensitivity and specificity for β‐gal with good fluorescence signal in the acidity range. Subsequently, this QMOH‐Gal probe was successfully employed to differentiate between normal cells and senescent cells by monitoring β‐gal. Furthermore, the probe not only realized the monitoring of β‐gal in zebrafish but also the tracking of β‐gal in palbociclib‐induced breast tumor senescence. Overall, the probe shows great promise as an effective tool for imaging β‐gal in vivo for studying the biology of aging in organisms.

## Graphical abstract

We develop a near‐infrared fluorescent probe for β‐gal tracking in lysosomes. The probe is successfully applied to distinguish senescent cells from non‐senescent cells. Importantly, the probe can track senescent cells in a mouse senescent tumor model.

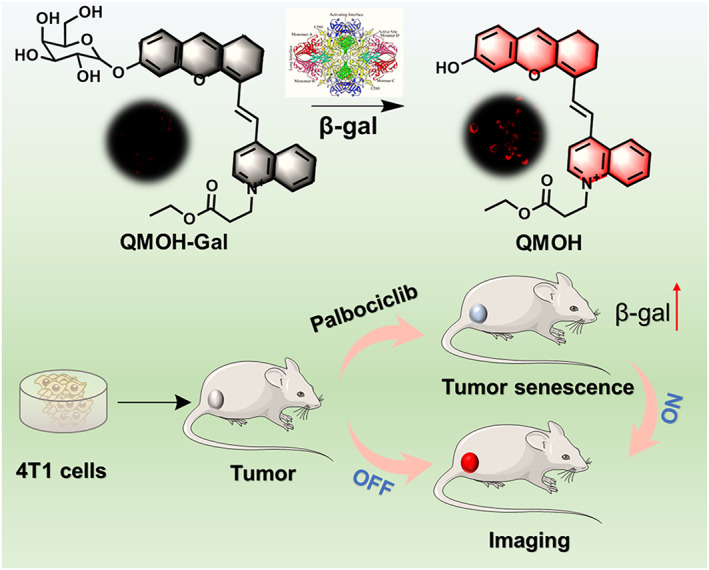

## 1 INTRODUCTION

As age increases, the functions of cells, tissues, and organs in the body decrease, which may lead to damage to the organism's immune system and further in turn causes an accumulation of senescent cells.[^1^, ^2^, ^3^] This accumulation of senescent cells can further impair tissue function and induce aging.[^4^, ^5^] In addition, senescent cells play key roles in a series of physiological and pathological processes, for instance, cancer, wound healing and remodeling, and other age‐related diseases.[^6^, ^7^] Moreover, senescence is recognized as an important factor in many chronic diseases, neurological disorders and the decline of human health.[^8^, ^9^, ^10^] Therefore, it is extremely urgent for early diagnosis and treatment of cellular senescence. Monitoring of disease‐related biomarkers can be effective in obtaining diagnostic and prognostic information, as well as in understanding the important role they play in the development of the disease.

As a crucial glycoside hydrolase in the body, β‐galactosidase (β‐gal) catalyzes the hydrolysis of a variety of glycoproteins and lactose.[^11^, ^12^] β‐Gal plays a significant role in multiple physiological processes and is closely linked with cellular senescence, which has been identified as one of its primary biological markers.[^13^, ^14^, ^15^, ^16^, ^17^] Therefore, monitoring β‐gal activity is of crucial importance, contributing to the early diagnosis of aging‐related diseases. Recently, several excellent strategies have been reported to detect β‐gal, including positron emission tomography, nuclear magnetic resonance, liquid chromatography‐mass spectrometry and fluorescence imaging techniques.[^18^, ^19^, ^20^, ^21^, ^22^] Fluorescent probes and their imaging techniques have received widespread attention because of their high resolution, selectivity, and non‐invasive real‐time imaging capabilities in cells and in vivo.[^23^, ^24^, ^25^, ^26^, ^27^, ^28^] Compared with fluorescent probes in the UV‐visible region, fluorescent probes in the near‐infrared (NIR) are more suitable for imaging biological tissues because of their high penetration ability, small light scattering and damage, and low background signal.[^29^, ^30^, ^31^, ^32^] In addition, β‐gal is mainly found in lysosomes with a pH of 4.5–5.5.[^33^, ^34^] Therefore, it is particularly important to develop a fluorescent probe that can have good properties under acidic conditions. However, there are limited reports of NIR fluorescent probes that have good biological properties under acidic conditions and are specific localized in lysosomes have been reported for β‐gal monitoring. Moreover, the majority of probes only detect β‐gal at the cellular level, and developing suitable for the detection of β‐gal remains a huge challenge in vivo.

In this work, we have developed a lysosome targeted near‐infrared fluorescent probe (QMOH Gal) for detecting β‐gal, which is stable in acidic pH. The introduction of a d‐galactose moiety rendered QMOH‐Gal essentially non‐fluorescent. In the presence of β‐gal, this QMOH‐Gal probe exhibited strong fluorescence emission at 740 nm. Furthermore, QMOH‐Gal demonstrated high selectivity and sensitivity towards β‐gal. This highly biocompatible probe can not only be effectively localized in lysosomes, but has also been successfully applied to differentiate between normal and senescent cells. QMOH‐Gal was also successfully applied in zebrafish for β‐gal detection. Importantly, the probe can track senescent cells in a drug‐induced mouse tumor model of senescence.

## 2 RESULTS AND DISCUSSION

### 2.1 Design and synthesis of the probe QMOH‐Gal

**Scheme 1:**
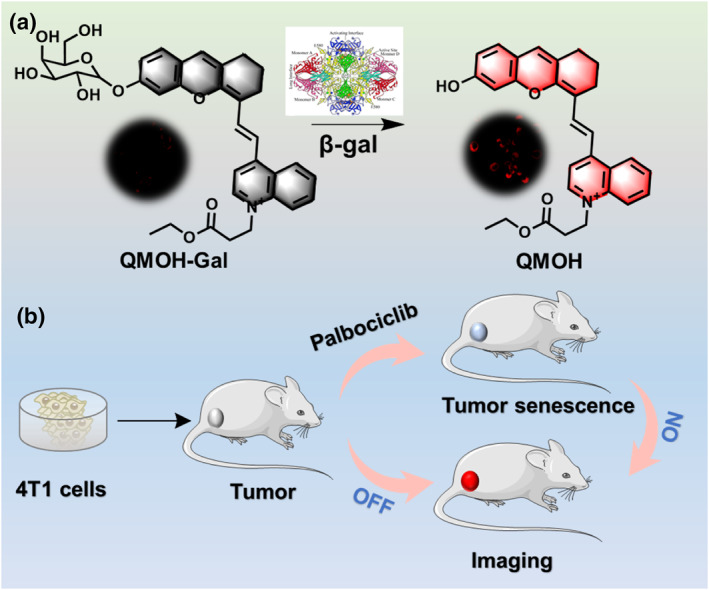
(a) The structure of this QMOH‐Gal probe and response mechanism to β‐gal. (b) The QMOH‐Gal probe for tracking cellular senescence in vivo.

The structure of the probe (QMOH‐Gal) and its response mechanism to β‐gal are shown in Scheme 1, and its synthesis route is shown in Figure S1. In this design, we introduced hemicarbocyanine as an NIR fluorophore due to its good biocompatibility and excellent spectral properties. To detect β‐gal sensitively and specifically, we introduced β‐d‐galactopyranoside as the response site of β‐gal. The presence of β‐d‐galactopyranoside causes the probe to exhibit weak fluorescence signals. When β‐gal was present, the fluorescence signal of the probe was turned on, releasing an obvious NIR fluorescence signal at 740 nm. In addition, we verified the successful synthesis of the probe by NMR and mass spectrometry (Figures S2–S8). The NMR and MS (*m*/*z* [C_35_H_38_NO_9_^+^], calcd: 616.2542; found [M]^+^: 616.2533) results proved that the probe QMOH‐Gal was successfully synthesized.

### 2.2 QMOH‐Gal response to β‐gal

**FIGURE 1:**
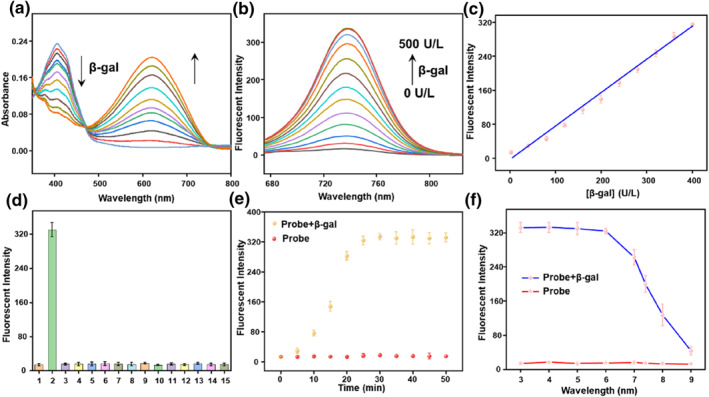
Spectral properties of the probe QMOH‐Gal (10 μM) to β‐gal in PBS containing 5% DMSO (pH = 7.4, 10 mM). (a) The ultraviolet‐visible absorption spectrum and (b) the fluorescence spectrum of QMOH‐Gal (10 μM) for β‐gal (0–500 U/L, *λ*_ex_ = 640 nm, slit widths: 5/5 nm). (c) The linear relationship between the fluorescence intensity of QMOH‐Gal and β‐gal (0–400 U/L). (d) The reaction of QMOH‐Gal to a series of various analytes: (1) blank, (2) β‐gal (500 U/L), (3) esterase (500 U/L), (4) ALP (500 U/L), (5) AFU (100 U/L), (6) Cys (1 mM), (7) Hcy (1 mM), (8) GSH (1 mM), (9) BSA (100 μg/ml), 10. HClO (100 μM), (11) NaNO_2_ (100 μM), (12) H_2_O_2_ (100 μM), (13) Cl^−^ (100 μM), (14) Na^+^ (100 μM), (15) K^+^ (100 μM). Fluorescence spectrum at 740 nm of QMOH‐Gal (e) with time change and (f) pH effect to β‐gal at 37°C.

After determining that the probe QMOH‐Gal was successfully synthesized, we further evaluated whether the probe could be used for β‐gal detection by UV–visible absorption spectroscopy and fluorescence spectroscopy in phosphate buffer saline solution containing 5% DMSO (pH = 7.4, 10 mM). As shown in Figure 1a, QMOH‐Gal showed a distinct absorption peak at around 415 nm. However, a new absorption peak appeared around 621 nm in the presence of β‐gal. Moreover, with the increase of β‐gal levels, the absorption peak at 415 nm gradually reduced and the absorption peak slowly increased at 621 nm, which was attributed to the reaction of β‐gal with β‐d‐galactopyranoside in the probe to produce a new substance. This further revealed that QMOH‐Gal could be applied for the monitoring of β‐gal. Subsequently, we further investigated the changes in the fluorescence signals of QMOH‐Gal. As shown in Figure 1b, QMOH‐Gal essentially exhibited a weak fluorescence signal. However, the fluorescence peak of the QMOH‐Gal probe at 740 nm was significantly enhanced with the addition of β‐gal, which should be attributed to the hydrolysis of the glycosidic bond by β‐gal, releasing the naked hydroxyl fluorophore (QMOH). Notably, there was an excellent linear relationship between the fluorescence intensity of QMOH‐Gal and the level of β‐gal (0–400 U/L). Furthermore, the associated linear relationship was assessed to be F_740 nm_ = 0.7874 [β‐gal] – 4.0995 with *R* ^2^ = 0.9937 (Figure 1c). The detection limit (LOD) was evaluated as 4.2 U/L via a standard method of 3σ/k. These results indicated that the probe had great potential for monitoring trace β‐gal.

### 2.3 Selectivity study of the probe QMOH‐Gal

Whether the probe can specifically detect β‐gal is also an important criteria for evaluating probes. Here, we have selected several common biological particles for evaluation, including enzymes (esterase, alkaline phosphatase (ALP), *α*‐l‐fucosidase (AFU)), biothiols (cysteine (Cys), homocysteine (Hcy), glutathione (GSH), tyrosine (Tyr), tryptophan (Try), methionine (Met), glycine (Gly), lysine (Lys), asparticacid (Asp)) and common ions (hypochlorous acid (HClO), hydrogen peroxide (H_2_O_2_), sodium nitrite (NaNO_2_), bovine serum albumin (BSA), adenosine triphosphate (ATP), Ca^2+^, Zn^2+^, Fe^2+^, Cl^−^, Na^+^, K^+^. As displayed in Figure 1d and Figure S9, only β‐gal induced a strong change in the fluorescence signal, whereas the other substances studied did not cause a significant change in the fluorescence signal. These results suggested that the probe could selectively respond to β‐gal without interference from other substances.

### 2.4 Response time and the pH effect toward QMOH‐Gal

Response time is also an important factor in evaluating the merits of a probe; we next tested the reaction time of QMOH‐Gal towards β‐gal at 37°C. As shown in Figure 1e, the fluorescence intensity of the QMOH‐Gal probe increased significantly in 5–20 min and reached a plateau after 25 min. The above experimental results exhibited that QMOH‐Gal could be capable of real‐time monitoring of β‐gal. We further investigated whether the probe could be used for prolonged detection of β‐gal without being affected. Here, we can observe that the fluorescence signal of QMOH‐Gal remains unchanged with the extension of time, indicating that the probe has good photostability and great potential to be employed to long‐time imaging of β‐gal in biological systems. Subsequently, to further investigate whether the QMOH‐Gal probe could be used for β‐gal detection under physiological conditions, the effect of pH on QMOH‐Gal was further studied. As shown in Figure 1f, the probe QMOH‐Gal was essentially devoid of fluorescence signal and remained stable over a pH range of 3‐9 in the absence of β‐gal, implying that the probe was not affected by pH. However, in the presence of β‐gal, the probe reached its maximum fluorescence intensity and remained stable at a pH of acidic. Since β‐gal is predominantly found in lysosomes, which have a pH of around 4.5–5.5, the probe could be applied for β‐gal monitoring in biological systems.

### 2.5 Lysosomal co‐localization studies

**FIGURE 2:**
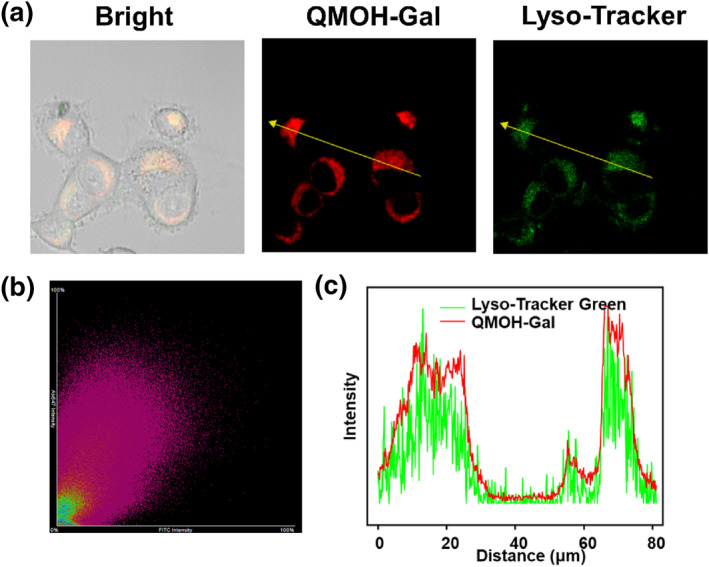
Lysosomal co‐localization studies of Lyso‐Tracker Green with QMOH‐Gal in Ovcar‐3 cells. (a) Fluorescence imaging of Lyso‐Tracker Green co‐incubated with the probe in Ovcar‐3 cells. (b) Correlation between the green and red regions crossed by the yellow arrows in (a). (c) Intensity distribution map of the green and red channels indicated by the yellow arrows in (a). Green channel: 500–560 nm (*λ*_ex_ = 488 nm) and red channel: 680–770 nm (*λ*_ex_ = 640 nm).

Previous studies have shown that β‐gal is predominantly found in the lysosomes of cells, so we tried to further investigate whether the probe could be localized in the lysosomes of living cells.^[35]^ Here, a commercially available lysosomal dye (Lyso‐Tracker Green) was co‐incubated with our probe for the study. As shown in Figure 2a, there was a very good overlap between the green fluorescent channel of Lyso‐Tracker Green and the red channel of QMOH‐Gal. In addition, a high degree of fluorescence synchronization between the lysosomal green probe and QMOH‐Gal was found by analyzing the yellow arrowed sites labeled in the green and red channels, whose Pearson's correlation coefficient was calculated to be 0.86 (Figures 2b,c). These results indicated that the QMOH‐Gal probe can efficiently aggregate in the lysosomes of cells.

### 2.6 Imaging β‐gal in senescent cells

**FIGURE 3:**
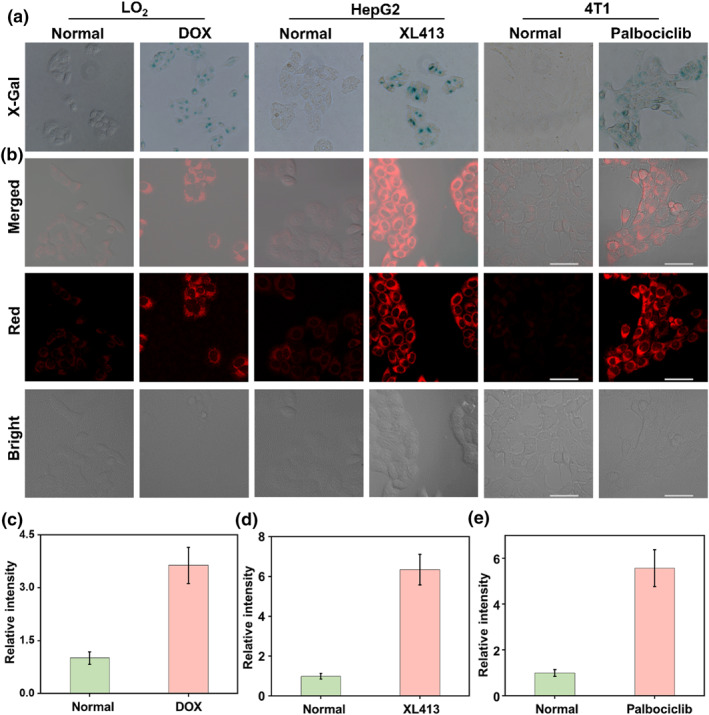
Visualization imaging of intracellular β‐gal. (a) X‐Gal staining of endogenous β‐gal in LO_2_ cells and DOX‐induced LO_2_ senescent cells, HepG2 cells and XL413‐induced HepG2 senescent cells, and 4T1 cells and palbociclib‐induced 4T1 senescent cells. (b) Fluorescence imaging of β‐gal with QMOH‐Gal in LO_2_ cells and DOX‐induced LO_2_ senescent cells, HepG2 cells and XL413‐induced HepG2 senescent cells, and 4T1 cells and palbociclib‐induced 4T1 senescent cells. (c) Relative fluorescent intensities in LO_2_ cells and DOX‐induced LO_2_ senescent cells. (d) Relative fluorescent intensities in HepG2 cells and XL413‐induced HepG2 senescent cells. (e) Relative fluorescent intensities in 4T1 cells and palbociclib‐induced 4T1 senescent cells. *λ*_ex_ = 640 nm, *λ*_em_ = 680–770 nm. Scale bars: 50 μm.

Encouraged by the excellent spectral properties of the probe, we attempted to use the probe QMOH‐Gal to track β‐gal in vivo. Prior to this, we first assessed the biotoxicity of the probe QMOH‐Gal through the cell counting kit‐8 (CCK‐8) experiment. As shown in Figure S10, the probe showed high survival of all three cells ((human normal hepatocytes (LO_2_ cells), human hepatocellular carcinoma cells (HepG2 cells), and mouse breast cancer cells (4T1 cells)) even at higher concentrations, indicating low biotoxicity and good biocompatibility of the probe. Subsequently, we investigated the ability of this QMOH‐Gal probe for intracellular monitoring of β‐gal. As shown in Figures 3a,b, we observed an essentially weak fluorescent signal in LO_2_ cells, implying that the amount of β‐gal in LO_2_ cells was low. Subsequently, we co‐incubated the probe with HepG2 cells or 4T1 cells. Here, we also found a weak red fluorescent signal, indicating the level of β‐gal was also low in both cells. Recent studies have shown that β‐gal is overexpressed in senescent cells compared to other cells.[^36^, ^37^] Therefore, we attempted to track β‐gal in senescent cells using this probe.

For LO_2_ cells, cellular senescence was induced by treatment with doxorubicin (DOX) for 5 days^[38]^ For HepG2 cells, we chose to treat them with XL413, which induces cellular senescence by inhibiting the function of the DNA replication kinase CDC7.^[37]^ For 4T1 cells, we selected palbociclib, a CDK4/6 inhibitor that inhibited DNA replication and induced cycle arrest in breast cancer cells or other cancer cells.[^39^, ^40^] Subsequently, we verified that the senescence model was successfully established by X‐gal (Figure 3a). Here, relative to the untreated cells, we could observe a distinct blue color in the drug‐treated cells, which indicated the successful establishment of the cellular senescence model. Next, we investigated the ability of the probe to be used to accurately diagnose cellular senescence. As shown in Figures 3b–e, bright red fluorescent signals were obtained in senescence cells, which not only demonstrated that the probe QMOH‐Gal could be used for β‐gal detection, but also implied that the level of β‐gal was relatively high in senescence cells. These results suggested that this QMOH‐Gal probe could be effectively applied for β‐gal monitoring in cells and could effectively distinguish senescence cells from other cell lines, which could contribute to the diagnosis of aging‐related diseases.

### 2.7 Imaging of β‐gal in zebrafish

**FIGURE 4:**
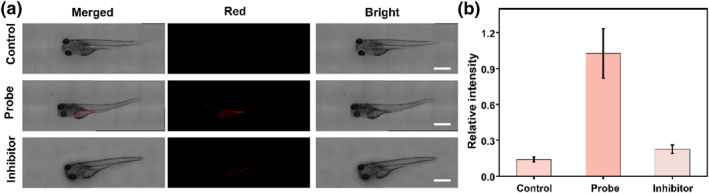
(a) Imaging of β‐gal with the QMOH‐Gal probe in zebrafish. Control group: Zebrafish do not undergo any treatment; probe group: the zebrafish was pre‐incubated with this probe QMOH‐Gal for 40 min and then imaged; inhibitor group: the zebrafish was stimulated with d‐galactoside for 60 min and then stained with the probe QMOH‐Gal for 40 min. (b) Relative fluorescent intensities in (a). *λ*_ex_ = 640 nm, *λ*_em_ = 680–770 nm. Scale bars: 500 μm.

To further investigate the ability of this probe QMOH‐Gal to monitor β‐gal in vivo, here, we chose zebrafish as a study subject. Previous research has proved that the β‐gal content in embryonic zebrafish is very low. However, β‐gal levels in the liver and yolk sac are significantly higher in zebrafish after 48 h.[^41^, ^42^] Therefore, we chose 4‐day‐old zebrafish as the study model. As shown in Figure 4, essentially no obvious fluorescence signal was observed in zebrafish without any treatment (control group). However, upon co‐incubation of the probe QMOH‐Gal with zebrafish, we could observe a clear red fluorescent signal (probe group), which revealed that this probe QMOH‐Gal could be employed to detect β‐gal in vivo. Moreover, we pre‐treated zebrafish with d‐galactoside (1 mM, a β‐gal inhibitor) for 60 min and then co‐incubated with the probe QMOH‐Gal for 40 min, and the red fluorescent signal disappeared (inhibitor group), indicating that QMOH‐Gal could be applied to detect fluctuations in β‐gal levels. Overall, the probe could be used as an effective tool for detecting and imaging fluctuations in exogenous and endogenous β‐gal levels in vivo.

### 2.8 The probe QMOH‐Gal for tracking of β‐gal in vivo

**FIGURE 5:**
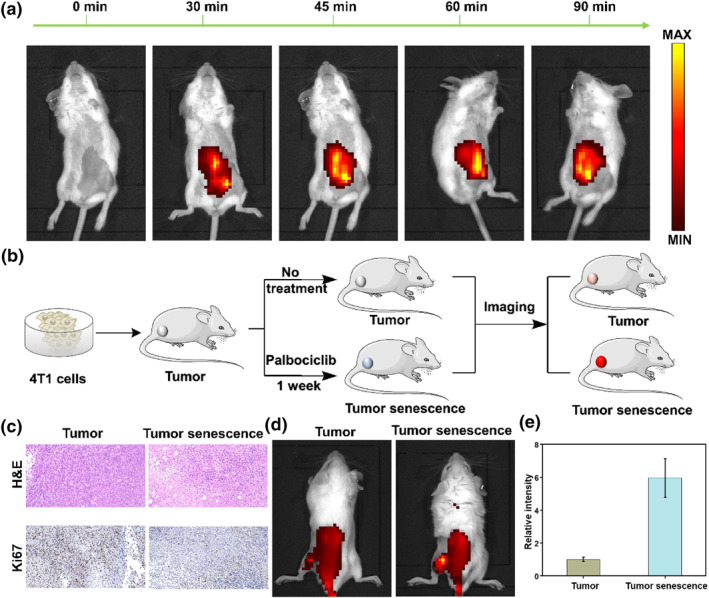
Imaging endogenous β‐gal in mice. (a) Imaging β‐gal in the mouse peritoneal cavity at different time points (0–90 min). (b) Schematic diagram of mouse tumor and senescence tumor modeling. (c) H&E and Ki67 staining for mouse tumors and palbociclib‐induced tumor senescence. (d) Imaging of β‐gal in mice tumor and senescence tumor modeling. (e) Relative fluorescence intensity in (d). *λ*_ex_ = 640 nm, *λ*_em_ = 680–770 nm.

Next, we investigated the probe's ability to visualize β‐gal in vivo. Firstly, we inject the probe QMOH‐Gal and β‐gal into the peritoneal cavity. As shown in Figure 5a, with the prolongation of time, the fluorescence intensity gradually increased, reaching its maximum value at 45 min and remaining stable, indicating that the probe could be used for the monitoring of β‐gal in vivo. To further investigate whether the probe could be applied for precise tracking of senescent cells. As shown in Figure 5b, we first established a 4T1 tumor mouse model and divided it into two groups. One group did not receive any treatment (tumor group), while the other group was treated with palbociclib to induce tumor aging (tumor senescence group), followed by in vivo imaging. We validated the successful establishment of the tumor model through hematoxylin and eosin (H&E) staining (Figure 5c). In addition, we performed Ki67 staining, which is the main marker of cell proliferation. Through Ki67 staining, we could observe that the tumors treated with palbociclib had significantly reduced Ki67, indicating the successful establishment of a tumor aging model. Subsequently, we conducted live imaging of mice. As shown in Figure 5d,e, compared to the tumor group, the tumor aging group induced by palbociclib showed stronger fluorescence signals, indicating that the probe can effectively visualize tumor aging in vivo. The above research results confirmed that the probe could be effectively used for precise tracking of β‐gal during the aging process, and may serve as an effective tool for detecting age‐related diseases.

## 3 CONCLUSION

In summary, we developed a novel lysosome NIR fluorescent probe (QMOH‐Gal) for sensitive and rapid monitoring of β‐gal, which exhibits excellent properties such as good biocompatibility, high selectivity and sensitivity, as well as a wide linear range. This probe can not only be used to distinguish senescent cells from other non‐senescent cells but also for visual tracking of β‐gal detection in zebrafish models. Importantly, the probe has also been successfully applied for the precise tracking of β‐gal in aging tumors, contributing to the diagnosis of age‐related diseases. We believe that the designed probe may serve as an effective tool for studying age‐related diseases.

## CONFLICT OF INTEREST STATEMENT

The authors declare no conflicts of interest.

## ETHICS STATEMENT

All animal experiments were performed according to the guidelines of the Care and Use of Laboratory Animals formulated by the Ministry of Science and Technology of China. All experimental protocols were approved by the Institutional Animal Care and Use Committee of Hainan Medical University.

## Supporting information

Supplementary Material

## Data Availability

The data that supports the findings of this study are available in the supplementary material of this article.
