# Supplementary material for: A β‐galactosidase activated near‐infrared fluorescent probe for tracking cellular senescence in vitro and in vivo
Source: Smart Mol. 2025 Jan 5;3(1):e20240062. doi: 10.1002/smo.20240062 (PMC12117884; doi:10.1002/smo.20240062)
Supplement: Supplementary file 1 — Supplementary Material [file SMO2-3-e20240062-s001.docx]

Supporting Information

A β-galactosidase Activated Near-infrared Fluorescent Probe for Tracking Cellular Senescence *In Vitro* and *In Vivo*

Tian Su^a,b†^, Ruijun Shen^c†^, Dengchu Tu^a,b^, Xiaoyue Han^d^, Xianzhu Luo^a,b,c *^, Fabiao Yu^a,b *^

^a^ Key Laboratory of Emergency and Trauma, Ministry of Education, Key Laboratory of Haikou Trauma, Key Laboratory of Hainan Trauma and Disaster Rescue, The First Affiliated Hospital of Hainan Medical University, Hainan Medical University, Haikou 571199, China.

^b^ Engineering Research Center for Hainan Bio-Smart Materials and Bio-Medical Devices, Key Laboratory of Hainan Functional Materials and Molecular Imaging, College of Emergency and Trauma, College of Pharmacy, Hainan Medical University, Haikou 571199, China.

^c^ Shanghai Engineering Research Center of Molecular Therapeutics and New Drug Development, Department of Chemistry, School of Chemistry and Molecular Engineering, East China Normal University, Shanghai 200241, China.

^d^ Ludwig Institute for Cancer Research, University of Oxford, Oxford, UK, OX37DQd.

^†^ Tian Su and Ruijun Shen contributed equally to this paper.

Corresponding Author Email: luoxianzhu@muhn.edu.cn; yufabiao@muhn.edu.cn

**1. Apparatus and Materials.**

The phosphorus tribromide (PBr_3_), N, N-dimethylthiocarbamoyl chloride, boron tribromide (BBr_3_), cesium carbonate (Cs_2_CO_3_), 4-methylsalicylaldehyde, cyclohexanone, propylene glycol, 4-fluoro-2-hydroxybenzaldehyde, 4-chlorosalicylalde were supplied from Anergy Corporation. D-galactoside and lipopolysaccharide (LPS) were obtained from Source Leaf Bio. ^1^H and ^13^C NMR spectra, UV–vis, fluorescence spectra and the images were acquired from Bruker Avance II NMR spectrometer (Germany), U-V-2450, F-7000 (Japan) and Olympus FV1000 (Japan), respectively.

**2. Synthesis of the main compounds.**

**Figure S1.** Synthesis route of compound QMOH-Gal.

**2.1 Synthesis of compound 2**

Stirred DMF (8 mL) and chloroform (50 mL) at 0 °C for 30 min, then added PBr_3_ solution (10 mL) dropwise to the mixture. After stirring for 60 min, cyclohexanone (4 mL) was added. Next, the reaction mixture was placed at room temperature for 12 h, and then poured into water, adjusted the pH value to neutral, and extracted with CH_2_Cl_2_. The crude product is directly used for the next step without purification.

**2.2 Synthesis of compound 3**

Under nitrogen protection, 2-hydroxy-4-methoxybenzaldehyde (0.76 g, 5 mmol), cesium carbonate (5 g, 15 mmol), and 2-bromo-1-cyclohexene-1-formaldehyde (0.9 g, 5 mmol) were dissolved in 5 mL anhydrous DMF at 25 °C and stirred overnight. After completion, pour the solution into water and extract with CH_2_Cl_2_. The crude product was purified by column chromatography (200-300 mesh) using a gradient of petroleum ether and methanol (5:1, v/v) to obtain a yellow solid. ^1^H NMR (500 MHz, CDCl_3_) δ 10.34 (s, 1H), 7.11 (d, *J* = 9.1 Hz, 1H), 6.74 – 6.65 (m, 3H), 3.87 (s, 3H), 2.59 (ddd, *J* = 7.7, 5.0, 1.5 Hz, 2H), 2.47 (t, *J* = 6.1 Hz, 2H), 1.74 (m, *J* = 6.2 Hz, 2H).

**2.3 Synthesis of compound 4**

Compound 3 (1.21g) was dissolved in 10 mL anhydrous CH_2_Cl_2_ under ice bath, and then add diluted BBr_3_ (2 mL, dissolved in 5 mL CH_2_Cl_2_) dropwise. After stirring at ice bath for 1 h, then the mixture reacted overnight at room temperature. Then slowly pour it into water to collect the solid. ^1^H NMR (500 MHz, DMSO-*d_6_*) δ 10.18 (s, 1H), 7.20 (d, *J* = 8.3 Hz, 1H), 6.94 (s, 1H), 6.66 – 6.59 (m, 2H), 2.54 (d, *J* = 5.8 Hz, 2H), 2.28 (t, *J* = 6.0 Hz, 2H), 1.60 (m, *J* = 6.1 Hz, 2H).

**2.4 Synthesis of compound 5**

Dissolved compound 4 (0.228 g, 1 mmol), tetraacetyl -α-D-bromogalactose (1.65 g, 2 mmol), anhydrous sodium sulfate (0.6 g, 4 mmol), and cesium carbonate (1.3 g, 4 mmol) in 6 mL of anhydrous DMF. After about 8 h, poured the solution into water to precipitate a yellow solid, filtered to obtain compound 5. The crude product is directly used for the next step without purification.

**2.5 Synthesis of Compound 6**

Dissolved compound 5 in a mixed solution of 2 mL CH_2_Cl_2_/MeOH, and then add a certain sodium methoxide. After stirring for 2 h, filtered to give compound 6. No purification required, directly used for the next step. ^1^H NMR (500 MHz, DMSO-*d_6_*) δ 10.24 (s, 1H), 7.32 (d, J = 8.5 Hz, 1H), 6.98 (d, J = 13.4 Hz, 2H), 6.84 (d, J = 8.6 Hz, 1H), 5.22 (s, 1H), 4.90 (d, J = 7.7 Hz, 2H), 4.69 (s, 1H), 4.55 (s, 1H), 3.71 (s, 1H), 3.63 (t, J = 6.2 Hz, 1H), 3.60 – 3.54 (m, 2H), 3.53 – 3.49 (m, 1H), 3.42 (d, J = 9.8 Hz, 1H), 2.57 (t, J = 6.0 Hz, 2H), 2.30 (t, J = 6.0 Hz, 2H), 1.70 – 1.59 (m, 2H).

**2.6 Synthesis of Compound 8**

Add 4-methylquinoline (1.43 g, 10 mmol) and 3-bromopropionic acid (1.51 g, 10 mmol) to a round bottom flask containing 50 mL of anhydrous acetonitrile. Reflux the mixture overnight, and a large number of solid precipitates, filtered and obtained compound 8. ^1^H NMR (500 MHz, DMSO-*d_6_*) δ 9.13 – 9.05 (m, 1H), 8.42 (d, J = 8.5 Hz, 1H), 8.22 (d, J = 8.6 Hz, 1H), 8.13 (t, J = 7.7 Hz, 1H), 7.95 (dd, J = 10.5, 6.5 Hz, 2H), 3.09 – 3.03 (m, 2H), 2.98 (m, 2H), 2.95 (s, 3H).

**2.7 Synthesis of the probe** **QMOH-Gal.**

In a nitrogen atmosphere, compound 6 (0.39 g, 1 mmol), compound 8 (0.24 g, 1.11 mmol) and piperidine were added in a three-necked flask with 10 mL anhydrous ethanol. The mixture was stirred vigorously and heated to reflux overnight. At the end of the reaction, the solvent was removed by spinning, and the crude product obtained was purified by column chromatography to obtain a blue solid in 37% yield. ^1^H NMR (500 MHz, DMSO-*d_6_*) δ 9.46-9.45 (d, J = 4.9 Hz, 1H), 8.58–8.51 (dd, J = 18.5, 8.5 Hz, 2H), 8.24–8.22 (dd, J = 16.4, 8.7 Hz, 1H), 8.07 – 8.01 (m, 2H), 7.68–7.67 (d, J = 5.2 Hz, 1H), 7.30–7.28 (d, J = 8.5 Hz, 2H), 6.98–6.93 (d, J = 18.8 Hz, 2H), 6.82–6.80 (dd, J = 8.4, 1.9 Hz, 1H), 5.18 (d, 3H), 4.90–4.89 (d, J = 7.7 Hz, 1H), 4.12-4.10 (t, J = 5.1 Hz, 1H), 3.72–3.71 (d, J = 2.4 Hz, 1H), 3.63 (d, *J* = 5.8 Hz, 1H), 3.58 – 3.54 (m, 2H), 3.50 – 3.47 (m, 2H), 3.42 (dd, *J* = 9.5, 3.0 Hz, 2H), 3.01 – 2.79 (m, 6H), 2.60 – 2.52 (m, 2H), 2.28–2.06 (t, *J* = 5.6 Hz, 2H), 1.60 (s, 3H). ^13^C NMR (125 MHz, DMSO) δ (ppm) 186.67, 160.51, 159.47, 152.77, 149.95, 135.55, 129.93, 129.23, 128.33, 127.65, 126.76, 122.73, 119.74, 115.60, 113.05, 112.06, 103.42, 101.24, 76.04, 73.69, 70.66, 68.44, 60.69, 31.60, 30.24, 29.43, 28.80, 23.69, 22.84, 21.62, 20.35, 20.16, 14.35, 11.26. HR-MS: C_35_H_28_NO_9_^+^, 616.2541; found [M], 616.2533.

**3. Cell culture and cytotoxicity testing.**

LO_2_ cells, HepG2 cells were incubated in DMEM medium containing 10% FBS and penicillin-streptomycin, while 4T1 cells were incubated in 1640 medium. All three cells were placed in a 95% environment at 37 ℃ with 5% CO_2_. The cytotoxicity of the probe QMOH-Gal was evaluated using the CCK-8 assay. HeLa cells were placed in a 96-well plate at a density of 5000 cells per well and cultured for 24 h. The cells were then washed three times with PBS, and different concentrations of the probe are added to the cells, with further culture for an additional 24 h. Afterward, 10 μL of the CCK-8 reagent was added to each well, and the plate was incubated for approximately 4 h. The absorbance is then measured at a wavelength of 450 nm using a microplate reader (Tecan, Austria).

**4. Fluorescence imaging in living cells and Zebrafish.**

For cell imaging experiments, cells were first incubated in confocal dishes for 24 h. The cells were rinsed 2-3 times with PBS, then the probe was added and co-cultured for approximately 40 min, rinsed again 2-3 times with PBS and imaged. Zebrafish were first cultured in E3 embryo medium in an incubator at 28.5 ℃. We selected four-day-old zebrafish for the study, with a fluorescence collection window of 690-770 nm and an excitation wavelength of 640 nm.

**5. Establishment of mouse models.**

All experimental protocols were approved by the Institutional Animal Care and Use Committee of Hainan Medical University (Haikou, China, HYLL-2023-006). Firstly, BALB/C female mice were injected with 4T1 cells by subcutaneous injection and tumors formed two weeks later. To induce senescence, 100 mg/kg of palbociclib dissolved in 50 mmol of sodium lactate (pH = 5) was administered orally daily for 7 days. Fluorescence images were then taken with an IVIS spectral imaging system and a fluorescence imager. And tumor tissues were collected for H&E and Ki67 tissue staining. The fluorescence collection window was 690-770 nm and excitation wavelength was 640 nm, respectively.


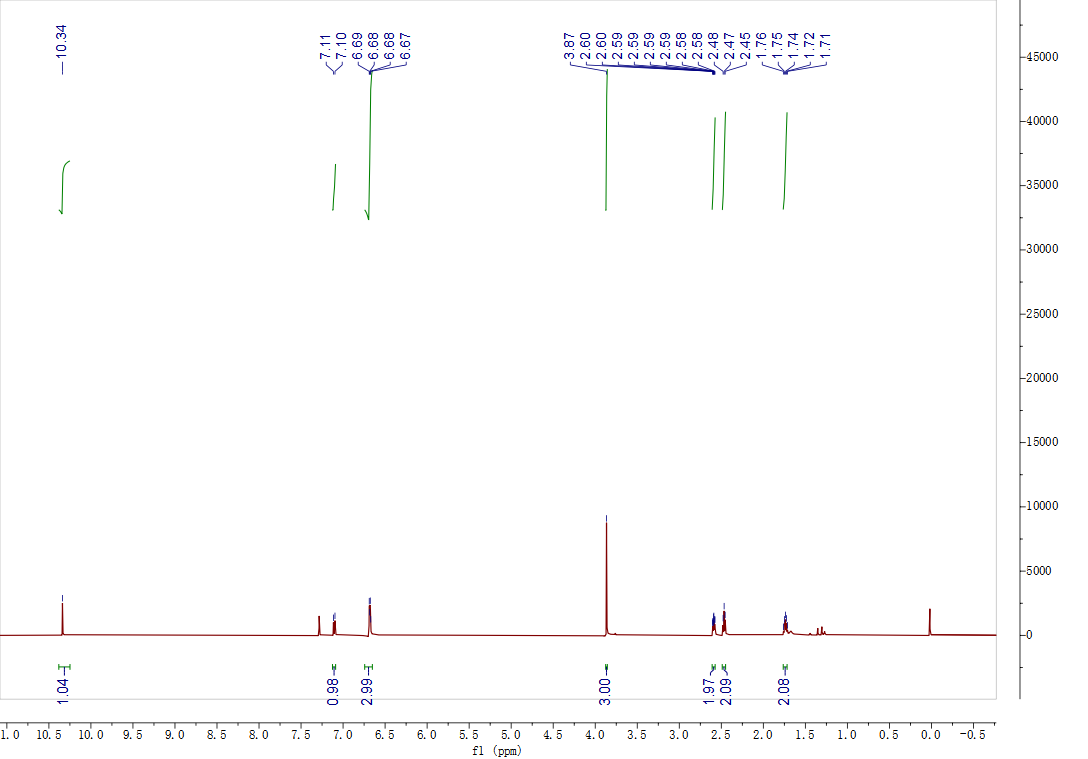


**Figure S2.** ^1^H NMR spectrum of compound 3 in CDCl_3_.


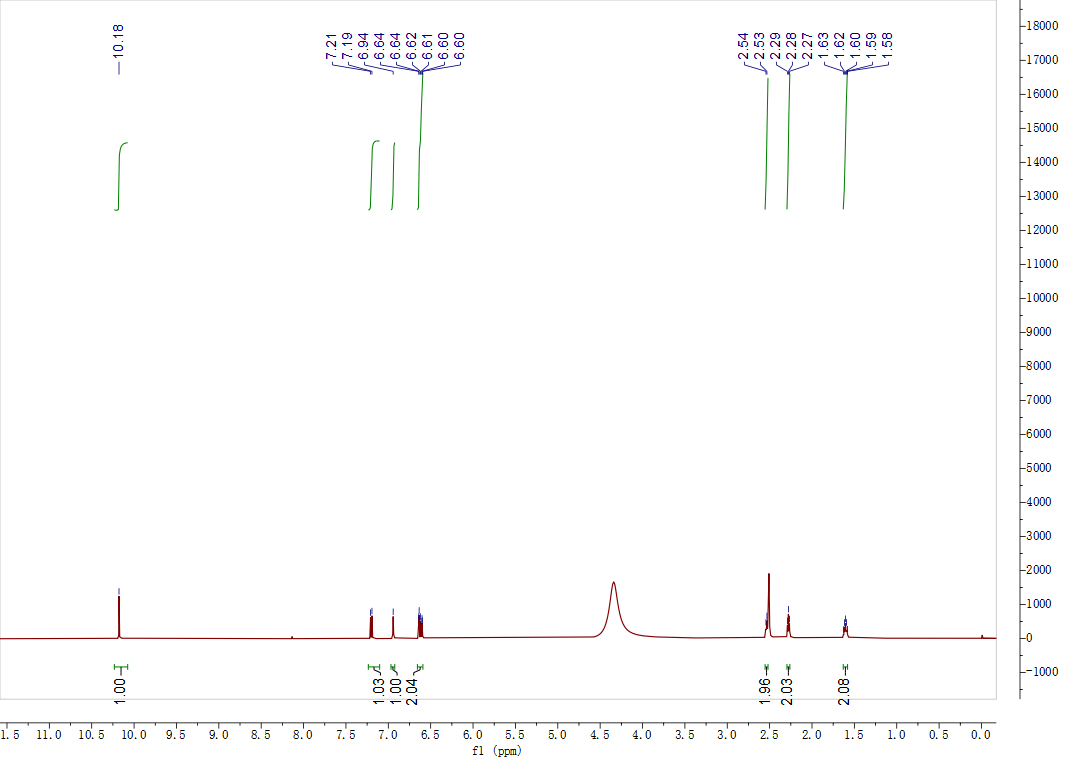


**Figure S3.** ^1^H NMR spectrum of compound 4 in DMSO-*d_6_*.


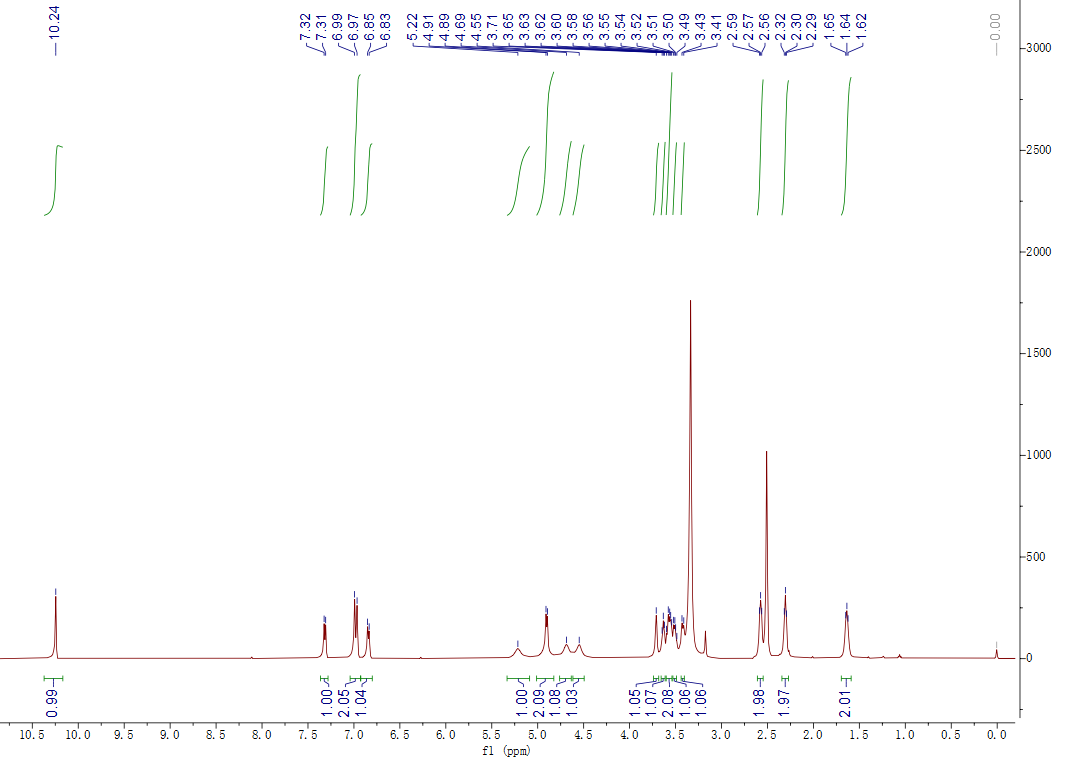


**Figure S4.** ^1^H NMR spectrum of compound 6 in DMSO-*d_6_*.


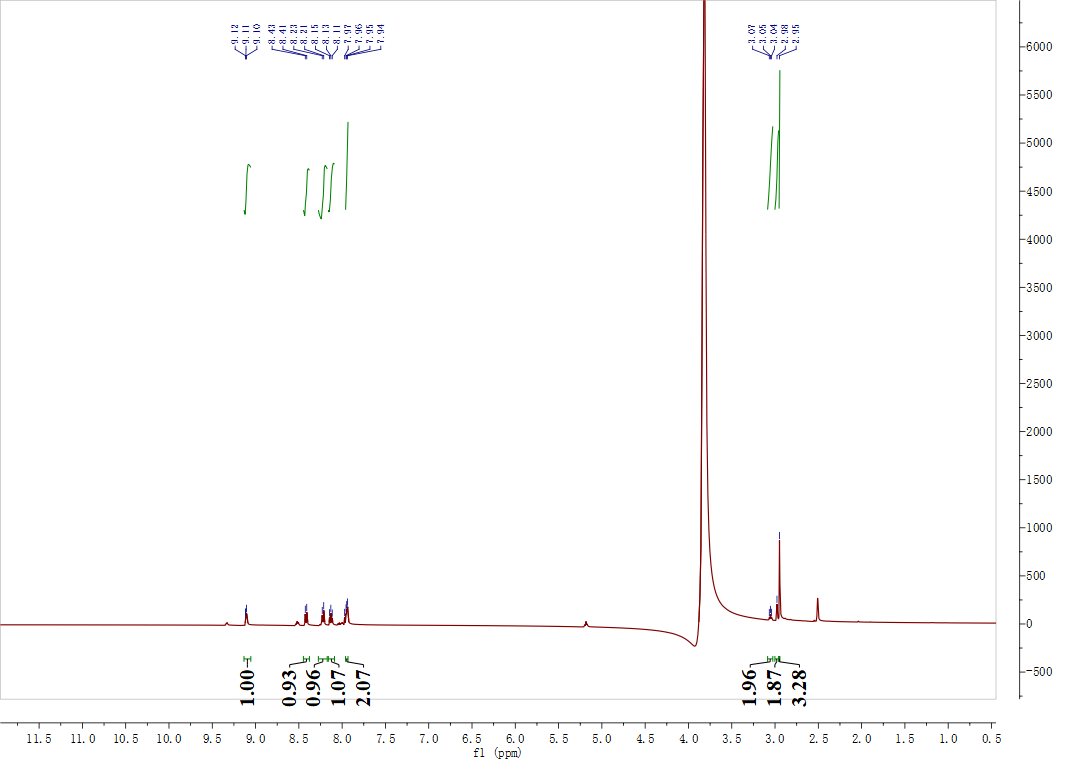


**Figure S5.** ^1^H NMR spectrum of compound 8 in DMSO-*d_6_*.


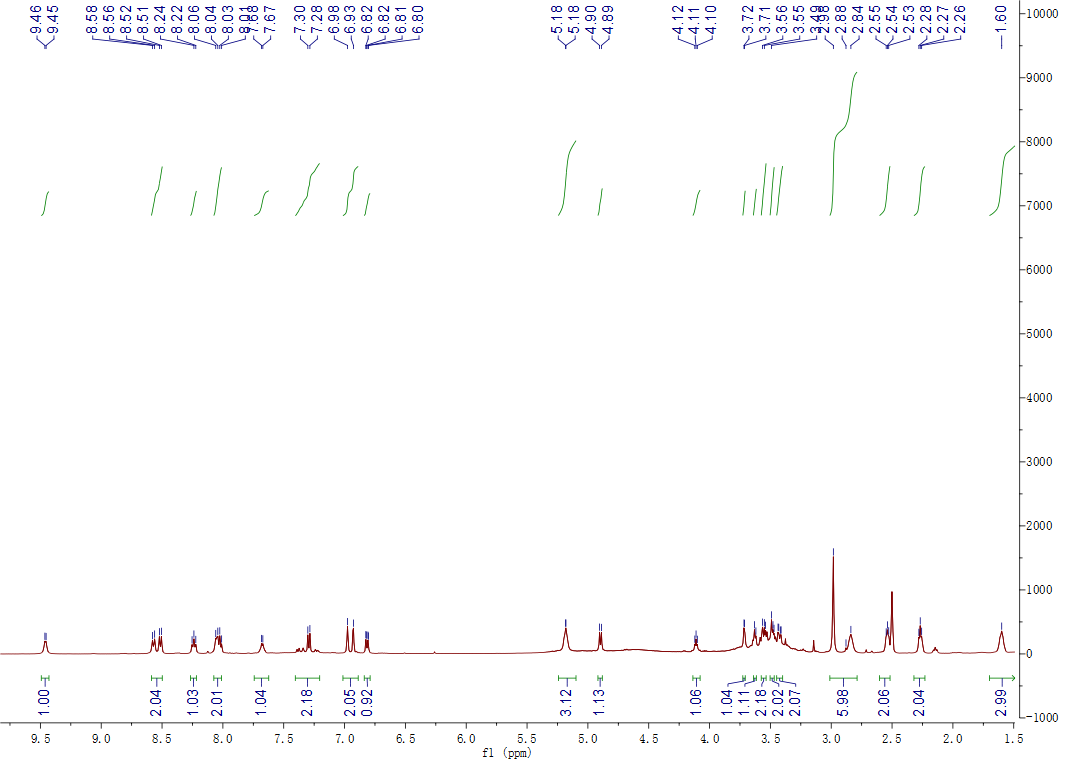


**Figure S6.** ^1^H NMR spectrum of QMOH-Gal in DMSO-*d_6_*.


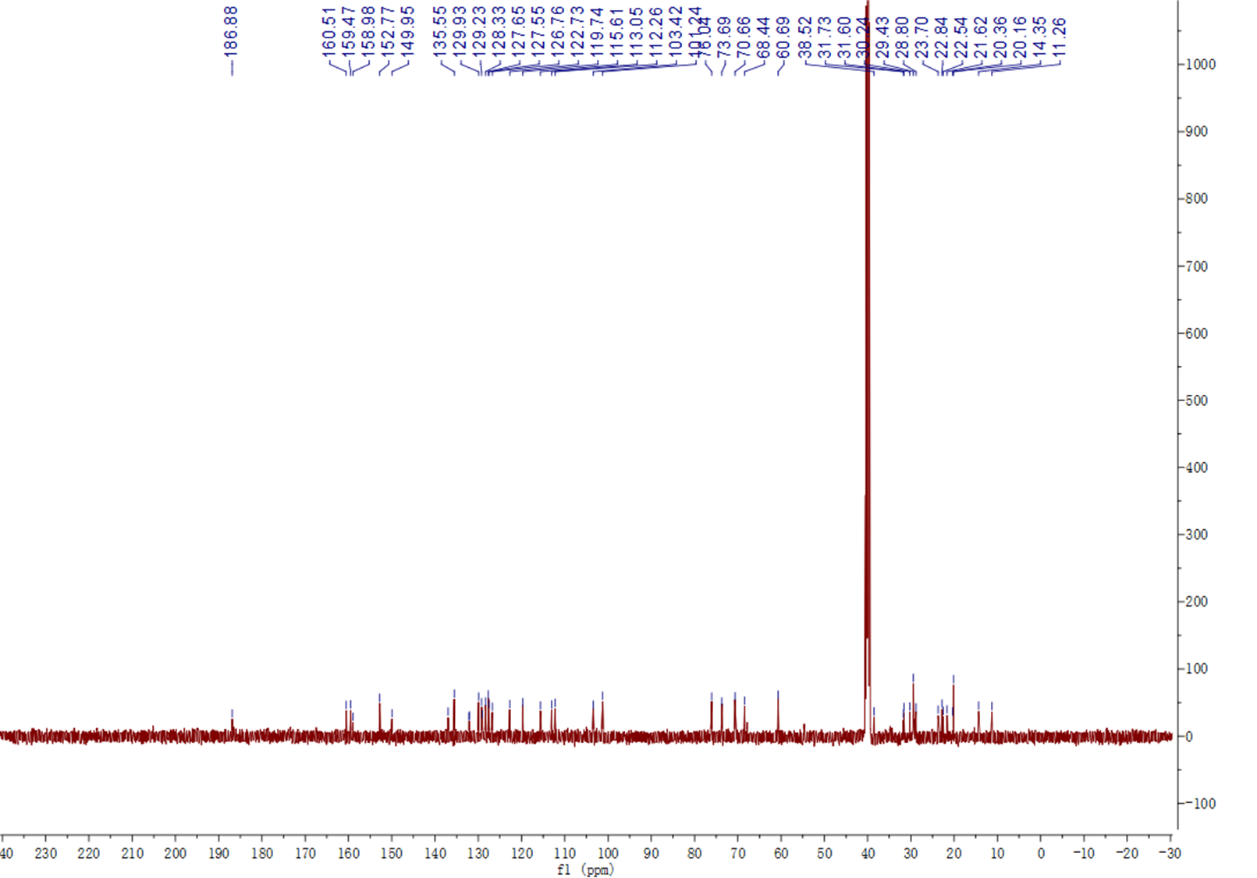


**Figure S7.** ^13^C NMR spectrum of QMOH-Gal in DMSO-*d_6_*.


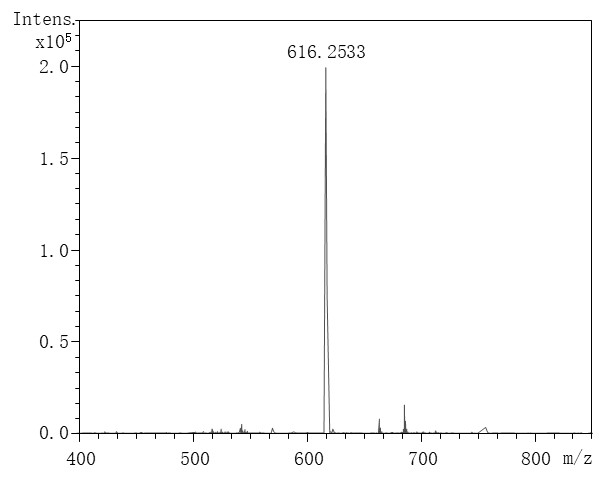


**Figure S8.** HR-MS of QMOH-Gal.


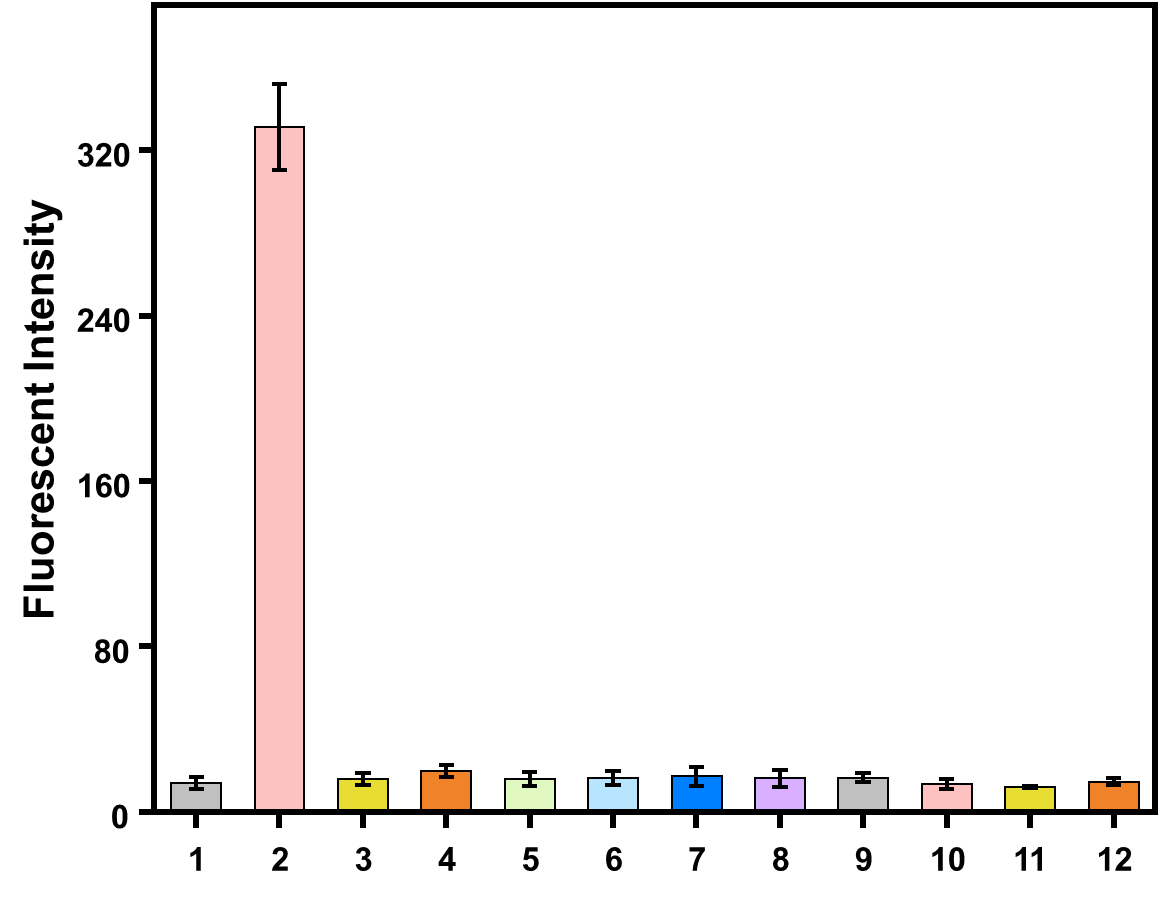


**Figure S9.** The reaction of QMOH-Gal to a series of various analytes: 1. blank; 2. β-gal (500 U/L), 3. ATP (100 µM), 4. Tyr (100 µM), 5. Trp (100 µM), 6. Met (100 µM), 7. Gly (100 µM), 8. Lys (100 µM), 9. Asp (100 µM), 10. Ca^2+^ (100 µM), 11. Zn^2+^ (100 µM), 12. Fe^2+^ (100 µM).


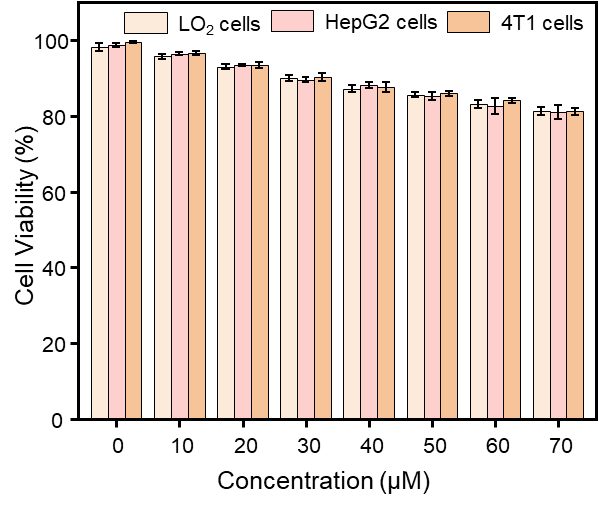


**Figure S10.** Cytotoxicity experiment of QMOH-Gal.
